# Meshfree Semi-Lagrangian Methods for Solving Surface Advection PDEs

**DOI:** 10.1007/s10915-022-01966-w

**Published:** 2022-08-22

**Authors:** Argyrios Petras, Leevan Ling, Steven J. Ruuth

**Affiliations:** 1 Johann Radon Institute for Computational and Applied Mathematics (RICAM), Altenbergerstrasse 69, 4040 Linz, Austria; 2 Hong Kong Baptist University, Kowloon Tong, Hong Kong; 3 Simon Fraser University, Burnaby, Canada

**Keywords:** Semi-Lagrangian method, Closest point method, Radial basis functions, Surface conservation laws, 65M06, 65M25

## Abstract

We analyze a class of meshfree semi-Lagrangian methods for solving advection problems on smooth, closed surfaces with solenoidal velocity field. In particular, we prove the existence of an embedding equation whose corresponding semi-Lagrangian methods yield the ones in the literature for solving problems on surfaces. Our analysis allows us to apply standard bulk domain convergence theories to the surface counterparts. In addition, we provide detailed descriptions for implementing the proposed methods to run on point clouds. After verifying the convergence rates against the theory, we show that the proposed method is a robust building block for more complicated problems, such as advection problems with non-solenoidal velocity field, inviscid Burgers’ equations and systems of reaction advection diffusion equations for pattern formation.

## Introduction

Partial differential equations (PDEs) arise frequently in the applied and natural sciences. In particular, conservation laws on surfaces can describe a variety of dynamics and mixing of surface intrinsic quantities, while preserving their initial concentrations. In some applications, the conservation laws do not include a diffusion term, or they appear to be advection dominated, with a velocity that is orders of magnitude larger than the coefficient of the diffusion.

Several methods are available to solve advection-dominated conservation laws on surfaces. These include the finite element method [3], the level set method [17] and the closest point method [15]. A typical approach when using finite elements is the inclusion of a stabilization term in order to avoid numerical instabilities. Such techniques include the streamline upwind Petrov-Galerkin method and the Galerkin least squares method [14]. Other stabilization techniques used in meshfree radial basis functions (RBF) methods include the hyperviscosity stabilization method, which has been applied on the solution of PDEs on surfaces using the RBF least orthogonal interpolation method [19].

In the class of the closest point method, advection dominated problems were numerically solved using an Eulerian approach and the RBF finite difference (FD) discretization in [12] by some total variation diminishing schemes, such as the TVD-RK methods [7]. When using standard finite differences, upwind schemes or centered differences have been used with the TVD-RK schemes [15]. Other numerical techniques use a semi-Lagrangian approach for solving advection dominated PDEs on static and moving surfaces, as well as the Navier-Stokes equation [1, 2]. However, the authors did not provide any convergence analysis of the methods or the conservation of the mass and momentum of the solution along time.

In this paper, we introduce a semi-Lagrangian numerical framework inspired by [2] for the numerical solution of advection dominated conservation laws. After providing proof of theoretical consistency with the surface PDE and numerical convergence analysis, the method is tested on a variety of examples on solenoidal velocity fields. Finally, a general framework is described that allows for the solution of rather general PDEs, including surface advection conservation laws with non-solenoidal velocity, strongly coupled systems and reaction-advection-diffusion PDEs.

## Advection Problems with Solenoidal Vector Field

Let $\varGamma \subset {\mathbb {R}}^d$ be a static surface embedded in ${\mathbb {R}}^d$ and $u:\varGamma \times {\mathbb {R}}^+\rightarrow {\mathbb {R}}$ be a scalar function. For some differentiable velocity field function ${\mathbf {v}}:\varGamma \times {\mathbb {R}}^+\rightarrow T_{\varGamma }\subset {\mathbb {R}}^d$ mapping any point on $\varGamma$ to the corresponding tangent space, the continuity equation can be written as

1 $$\begin{aligned} \left\{ \begin{array}{r@{~=~}l} \partial _t u + \nabla _\varGamma \varvec{\cdot }({\mathbf {v}}u) &{} 0, \\ u(\cdot , 0) &{} u_0 , \end{array} \right. \quad \text{ on } \varGamma . \end{aligned}$$

Further assume the velocity field ${\mathbf {v}}$ in (1) is solenoidal

$$\begin{aligned} \nabla _\varGamma \varvec{\cdot }{\mathbf {v}}=0. \end{aligned}$$

Then we have

2 $$\begin{aligned} \left\{ \begin{array}{r@{~=~}l} \partial _t u + {\mathbf {v}}\varvec{\cdot }\nabla _\varGamma u &{} 0, \\ u(\cdot , 0) &{} u_0, \end{array} \right. \quad \text{ on } \varGamma , \end{aligned}$$

using the product rule. Let ${\mathbf {x}}_0$ be some fixed point on $\varGamma$ and let $u({\mathbf {x}}_0,0)=u_0({\mathbf {x}}_0)$ be the initial solution of (2) at the point ${\mathbf {x}}_0$. Our goal is to identify the solution $u({\mathbf {z}}(t),t)$ along the characteristic curve ${\mathbf {z}}(t)$ that starts from the initial point ${\mathbf {x}}_0$ satisfying (2), see [8, 18]. In order to use the method of characteristics, which is a method for bulk domain type PDEs, a careful embedding should be derived.

### Theorem 1

Let $\varGamma$ be a closed $C^2$-surface, ${\mathbf {n}}={\mathbf {n}}({\mathbf {x}})$ be the unit outward pointing normal vector at ${\mathbf {x}}\in \varGamma$, and $u:\varGamma \rightarrow {\mathbb {R}}$ be the solution to (2). Then, there exists a tubular neighborhood $\varOmega \supset \varGamma$ of $\varGamma$ and an extended velocity field function ${\mathbf {v}}_\varOmega :\varOmega \rightarrow {\mathbb {R}}^d$ (i.e., ${\mathbf {v}}_{\varOmega }({\mathbf {x}}) ={\mathbf {v}}({\mathbf {x}})$ for all ${\mathbf {x}}\in \varGamma$) such that the constant-along-normal extension $u_\varOmega :\varOmega \rightarrow {\mathbb {R}}$ of the solution

$$\begin{aligned} u_\varOmega ({\mathbf {z}}, t) = u( cp_\varGamma ({\mathbf {z}}), t) \quad \text {for } {\mathbf {z}}\in \varOmega , \end{aligned}$$

solves the embedded PDE

3 $$\begin{aligned} \left\{ \begin{array}{ll} {\partial _t u_\varOmega } + {\mathbf {v}}_\varOmega \varvec{\cdot }\nabla u_\varOmega =0, &{} \text {in }\varOmega , \\ u_\varOmega (\cdot ,0) =(u_0 \circ cp_\varGamma ) , &{} \hbox { in}\ \varOmega , \\ \end{array}\right. \end{aligned}$$

where $cp_\varGamma :\varOmega \rightarrow \varGamma$ is the closest point mapping to the surface $\varGamma$.

### Proof

Since the surface $\varGamma$ is closed and smooth, there exists a tubular neighborhood $\varOmega$ of $\varGamma$ by the theories in [11]. Following [6] and by construction of $u_\varOmega$, the system (3) is well posed. Thus, using the method of characteristics, the solution $u_\varOmega$ must be constant along each path ${\mathbf {z}}(t)$ defined as

4 $$\begin{aligned} \begin{array}{l} \frac{d}{dt} {\mathbf {z}}(t)= {\mathbf {v}}_\varOmega ({\mathbf {z}}(t),t) \\ {\mathbf {z}}(0) = {\mathbf {z}}_0, \end{array} \end{aligned}$$

where ${\mathbf {z}}_0$ is the initial location of the solution $u_\varOmega$ at time 0. Since the $\varOmega$ is a tubular neighborhood of $\varGamma$, there exists a $C^1$-smooth closest point mapping $cp_\varGamma$ that maps points from the domain $\varOmega$ to their closest points [11]. Using the closest point mapping, we can write

$$\begin{aligned} {\mathbf {z}}(t) = cp_\varGamma ({\mathbf {z}}(t)) + \varPhi {\mathbf {n}}(cp_\varGamma ({\mathbf {z}}(t))), \end{aligned}$$

where $cp_\varGamma :\varOmega \rightarrow \varGamma$ is the closest point mapping from the embedding space to the surface $\varGamma$, and $\varPhi$ is the corresponding signed distance function. Substituting in (4) yields

$$\begin{aligned} {\mathbf {v}}_\varOmega ({\mathbf {z}}(t),t)= & {} \frac{d}{dt} (cp_\varGamma ({\mathbf {z}}(t))) + \frac{d}{dt}(\varPhi {\mathbf {n}}(cp_\varGamma ({\mathbf {z}}(t))))\\= & {} {\mathbf {v}}(cp_\varGamma ({\mathbf {z}}(t))) + \varPhi \frac{d}{dt}({\mathbf {n}}(cp_\varGamma ({\mathbf {z}}(t)))), \end{aligned}$$

which is a well-defined extension wherever $cp_\varGamma$ is differentiable. $\square$

Given that the initial condition is not a characteristic, see [6], we can solve (2) by applying the method of characteristics to the embedded PDE in (3) via the following system of ordinary differential equations (ODEs)

5 $$\begin{aligned} \left\{ \begin{array}{l} \frac{d}{dt}{\mathbf {z}}(t) = {\mathbf {v}}_\varOmega ({\mathbf {z}}(t),t), \\ {\mathbf {z}}(0) = {\mathbf {z}}_{0} \in \varOmega , \\ u_\varOmega ({\mathbf {z}}(t),t) = u_0(cp_\varGamma ({\mathbf {z}}_0)). \end{array}\right. \end{aligned}$$

Here, ${\mathbf {z}}(t)$ is the characteristic curve that starts from some point ${\mathbf {z}}_0\in \varOmega$ and ${\mathbf {v}}_\varOmega$ is the extended velocity defined in Theorem 1.

## Semi-Lagrangian Methods and Implementation on Point Clouds

Consider the characteristic curve ${\mathbf {z}}(t)$ of the embedded PDE (3) in $\varOmega$ that starts from a surface point ${\mathbf {x}}_0\in \varGamma \subset \varOmega$, say, at time $t=0$. Using the standard method of characteristics, we know that the solution $u_\varOmega$ must be constant along the characteristic curve ${\mathbf {z}}(t)$, which satisfies

$$\begin{aligned} \frac{d}{dt}{\mathbf {z}}(t) = {\mathbf {v}}_\varOmega \left( {\mathbf {z}}(t),t\right) . \end{aligned}$$

Thus, we must have

$$\begin{aligned} u_\varOmega ({\mathbf {z}}(t),t) = u_\varOmega ({\mathbf {x}}_0,0) \end{aligned}$$

and, since $u_\varOmega$ is constant along the normal direction by construction,

$$\begin{aligned} u_\varOmega ({\mathbf {z}}(t),t) = u_\varOmega (cp_\varGamma ({\mathbf {z}}(t)),t) . \end{aligned}$$

Thus, ${\mathbf {z}}(t)=cp_\varGamma ({\mathbf {z}}(t))\in \varGamma$ is a characteristic curve. Restricting the extended velocity field back to the surface yields an identity map, i.e.,

$$\begin{aligned} {\mathbf {v}}_\varOmega \left( cp_\varGamma ({\mathbf {z}}(t)),t\right) = {\mathbf {v}}\left( cp_\varGamma ({\mathbf {z}}(t)),t\right) . \end{aligned}$$

By applying the method of characteristics to an equivalent form of (3), we can rewrite ODE (5) as

6 $$\begin{aligned} \left\{ \begin{array}{l} \frac{d}{dt}{\mathbf {z}}(t) = {\mathbf {v}}\left( cp_\varGamma ({\mathbf {z}}(t)),t\right) , \\ {\mathbf {z}}(0) = {\mathbf {x}}_{0} \in \varGamma ,\\ u_\varOmega ({\mathbf {z}}(t),t) = u_{0}({\mathbf {x}}_0), \end{array}\right. \end{aligned}$$

for all $t>0$. Theorem 1 ensures the analytical solution (6) solves the surface advection problem (2) in Lagrangian coordinates.

Numerically, the bulk domain ODE system (6) can then be solved using any standard discretization scheme, in which the use of the closest point mapping allows for the use of quantities defined only on the surface. If we start at some initial data ${\mathbf {x}}^{n}\in \varGamma$ and apply one time step of an *s*-stage explicit Runge-Kutta method to the ODE (6), we arrive at a scheme with some extra internal projections

7 $$\begin{aligned} \left\{ \begin{array}{l} {\mathbf {k}}_i = {\mathbf {v}}\left( cp_\varGamma \left( {\mathbf {x}}^n + \varDelta t\sum \limits _{j=1}^{i-1}a_{ij}{\mathbf {k}}_j\right) ,t_n+c_i\varDelta t\right) , \quad ~i=1,\ldots ,s,\\ {\mathbf {x}}^{n+1} = {\mathbf {x}}^n + \varDelta t\sum \limits _{j=1}^s b_j {\mathbf {k}}_j, \end{array} \right. \end{aligned}$$

where $t_n$ is the time, the superscript of ${\mathbf {x}}^{\varvec{\cdot }}$ is the step of the temporal discretization with step-size $\varDelta t$, and the coefficients $a_{ij}$, $b_j$ and $c_i$ define the RK scheme.

Generally speaking, we know that ${\mathbf {x}}^{n+1}\not \in \varGamma$ from scheme (7) due to numerical error. To correct this, we want to further project this new approximation point ${\mathbf {x}}^{n+1}$ back to the surface, so that the result is in agreement with the analytical property of the solution of the embedded PDE (3). This leads us to introduce the closest point mapping into the second equation in (7) yielding a *new* scheme

8 $$\begin{aligned} \left\{ \begin{array}{l} {\mathbf {k}}_i = {\mathbf {v}}\left( cp_\varGamma \left( {\mathbf {x}}^n + \varDelta t\sum \limits _{j=1}^{i-1}a_{i,j}{\mathbf {k}}_j\right) ,t_n+c_i\varDelta t\right) \quad ,~i=1,\ldots ,s,\\ {\mathbf {x}}^{n+1} = cp_\varGamma \left( {\mathbf {x}}^n + \varDelta t\sum \limits _{j=1}^s b_j {\mathbf {k}}_j\right) , \end{array} \right. \end{aligned}$$

which is being used in [8, 18].

To see that the extra $cp_\varGamma$ in (8) does not impair the consistency of the method, let

$$\begin{aligned} {\mathbf {w}} = {\mathbf {x}}^n + \varDelta t\sum _{j=1}^s b_j {\mathbf {k}}_j \in \varOmega \end{aligned}$$

be some (possibly) out-of-surface solution to (7). Then, $cp_\varGamma ({\mathbf {w}})$ is the corresponding solution to the new problem (8). By the minimization property of $cp_\varGamma$ and the fact that the analytic solution ${\mathbf {x}}(t+\varDelta t)\in \varGamma$, an error estimate follows

$$\begin{aligned} \begin{aligned} \Vert cp_\varGamma ({\mathbf {w}}) - {\mathbf {x}}(t+\varDelta t)\Vert&= \Vert cp_\varGamma ({\mathbf {w}}) - {\mathbf {w}} + {\mathbf {w}} - {\mathbf {x}}(t+\varDelta t)\Vert , \\&\le \Vert cp_\varGamma ({\mathbf {w}}) - {\mathbf {w}}\Vert + \Vert {\mathbf {w}} - {\mathbf {x}}(t+\varDelta t)\Vert , \\&\le 2\Vert {\mathbf {w}} - {\mathbf {x}}(t+\varDelta t)\Vert . \end{aligned} \end{aligned}$$

**Fig. 1:**
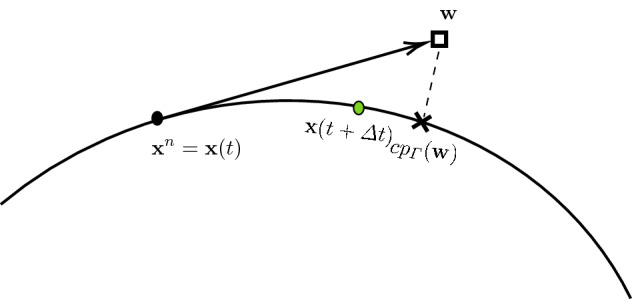
An illustration of the stepping procedures (7) and (8) using a forward Euler approximation. For the local error, assume time step $n+1$ starts from ${\mathbf {x}}^{n}= {\mathbf {x}}(t)$ (solid circle). Then, the bulk domain type RK solution ${\mathbf {w}}$ (square) is the approximated point using the standard scheme (7), while ${\mathbf {x}}^{n+1}=cp_\varGamma ({\mathbf {w}})$ (cross) is the approximation using scheme (8). Both ${\mathbf {w}}$ and our surface-restricted RK solution $cp_\varGamma ({\mathbf {w}})$ are within a distance $O(\varDelta t^2)$ of the exact ${\mathbf {x}}(t+\varDelta t)$ (green dot) (Color figure online)

The last inequality follows from the minimization property of $cp_\varGamma ({\mathbf {w}})$. In other words, the numerical error in (8) is at most twice as large as that in (7). This is the theoretical justification to the common sense approach in (8). An illustration of the stepping procedures (7) and (8) using a forward Euler approximation is provided in Fig. 1. We sum up our discussion with a theorem.

### Theorem 2

Suppose the assumptions in Theorem 1 hold. Then, the surface-restricted RK scheme (8) converges at the same rate as its standard bulk domain type counterpart.

We remarked that other numerical time integrators, i.e. linear multistep methods, can also be used with on-surface quantities only and a similar argument can be employed to show consistency via the embedded Eq. (3).

We point out that (8) requires the use of the $cp_\varGamma$ operator as ${\mathbf {v}}$ is only defined on $\varGamma$. Thus, no extension of the velocity outside the surface is required, since the closest point mapping projects the points back on the surface at each stage of the RK method.

### Algorithm for Solving Advection Eq. (2)

Let *Y* be a set of data points on the surface, i.e. $Y:=\{{\mathbf {y}}_j\}_{j}\subset \varGamma$. Suppose at some time $t_n\ge 0$, we know the solution values $\{ u_\varOmega ({\mathbf {y}}_j,t_n)\}_j$ for all ${\mathbf {y}}_j \in Y$. The aim is to update all nodal solution values to $\{ u_\varOmega ({\mathbf {y}}_j,t_{n+1})\}_j$ at time $t_{n+1}:=t_n+\varDelta t$ for some time step $\varDelta t>0$. The proposed numerical framework to solve the characteristic ODEs in (5) uses the following steps:

- **Identify the backtraced points:** For each surface point ${\mathbf {y}}_j\in Y$, solve the ODE
  9 $$\begin{aligned} \left\{ \begin{array}{l} \frac{d}{dt}cp_\varGamma ({\mathbf {z}}(t)) = {\mathbf {v}}(cp_\varGamma ({\mathbf {z}}(t)),t), \\ {\mathbf {z}}(t_{n+1}) = {\mathbf {y}}_j, \end{array} \right. \end{aligned}$$
  by the surface-restricted RK in (8) and approximate $cp_\varGamma$ as in Sect. 3.2 (if not known) in the time interval $[t_n,t_{n+1}]$ to approximate the backtraced points $\tilde{{\mathbf {z}}}(t_{n})$ and store $cp_\varGamma (\tilde{{\mathbf {z}}}(t_{n}))=:{\mathbf {x}}_j$.
- **Interpolate the solution on the backtraced points:**
  Using existing nodal solution values $\{( {\mathbf {y}}_j,\, u_\varOmega ({\mathbf {y}}_j,t_n) )\}_j$ as data and the RBF interpolation scheme in Sect. 3.3, interpolate $u_\varOmega$. Store the interpolated value as ${\tilde{u}}_\varOmega ({\mathbf {x}}_j)$.
- **Update the nodal solution:** Set $u_\varOmega ({\mathbf {y}}_j,t_{n+1}) := {\tilde{u}}_\varOmega ({\mathbf {x}}_j)$.

Note that Alg.1(c) identifies the solution at the initial point cloud *Y* at time $t_{n+1}$. Thus, there are no alterations introduced to the initial distribution of points after each time step.

### Computing Closest Points on Point Clouds

For parametrized, implicit, or triangulated surfaces, the closest point mapping can be calculated using the techniques described in [12, Sect.2.1.1]. However, for more general point clouds, the identification of the closest point mapping is not a straightforward task. We now describe a framework for finding the closest points on the surface based on some given oriented point clouds $X\subset \varGamma$.

Let ${\mathbf {z}}\in \varOmega$ be a point in the embedding space, which could be the resulting approximated location after any intermediate step in the RK method. Our goal is to approximate $cp_\varGamma ({\mathbf {z}})$. To do so, we propose to use a local surface reconstruction procedure, which is a variation of the resampling step of the grid based particle method in [10], to map the points in the embedding space to their closest points on the surface.

We aim to identify the *k* closest surface points *with proper orientation* to the out-of-surface point ${\mathbf {z}}\in \varOmega$. Suppose that *X* is sufficiently dense with respect to the stencil size *k*. One can easily collect a subset of $X_k({\mathbf {z}}):=\{{\mathbf {x}}_i\}_{i=1}^k\subset X$ by the *k* nearest points to ${\mathbf {z}}$. We avoid selecting any across-surface points with sufficient separating distances by enforcing two criteria: points in $X_k({\mathbf {z}})$ need to be

1. on the same surface segment, and
2. numerically distinct up to some user defined tolerance.

Let ${\mathbf {x}}_0=\min _{{\mathbf {x}}\in X}\Vert {\mathbf {x}}-{\mathbf {z}}\Vert$ be the closest point from the whole point cloud to the point ${\mathbf {z}}$, and ${\mathbf {n}}_0=n({\mathbf {x}}_0)$ its unit normal vector. Using the normal information of the point cloud, i.e., ${\mathbf {n}}_i := {\mathbf {n}}({\mathbf {x}}_i)$ for all ${\mathbf {x}}_i\in X$ with $1\le i\le k$, the first criterion (C1) is met by applying a normal continuity condition

10 $$\begin{aligned} \cos ^{-1}({\mathbf {n}}_i\varvec{\cdot }{\mathbf {n}}_0) =: \theta _i < \theta _{\max } \end{aligned}$$

for some user defined threshold angle $\theta _{\max }$.

Next, in (C2), we make sure the *k* collected points are well separated by enforcing

$$\begin{aligned} \min \{ \Vert {\mathbf {x}}_p - {\mathbf {x}}_q\Vert _2\,:\,{\mathbf {x}}_p, {\mathbf {x}}_q\in X_k({\mathbf {z}}) ,\; p\ne q\} > \delta _{\min }, \end{aligned}$$

where $\delta _{\min }$ is a specified threshold separating distance.

**Note:** If the point ${\mathbf {z}}$ in the embedded space is a result of an intermediate RK step, then ${\mathbf {x}}_0$ should lie on the same segment as the point before the motion, i.e., the point ${\mathbf {y}}$ in step Alg.1(a). This introduces another Lagrangian consistency condition for the unit normal vector continuity, similar to the one defined by Eq. (10).

At this stage, we have a stencil of on-surface points $X_k({\mathbf {z}})$ around the to-be-determined point $cp_\varGamma ({\mathbf {z}})$.

We now focus our discussion on 2D surfaces in ${\mathbb {R}}^3$; generalization to other dimensions is straightforward. Define a rotation operator ${\mathcal {R}}: {\mathbb {R}}^3 \rightarrow {\mathbb {R}}^3$ to rotate the unit normal vector ${\mathbf {n}}_0\in {\mathbb {R}}^3$ to the (positive) *z*-axis in 3D. Let ${\mathcal {R}}X_k({\mathbf {z}}) :=\{{\mathcal {R}}{\mathbf {x}}_i \}_{i=1}^k$ be the set of rotated stencil. We use their (a.k.a. local) coordinates of the point ${\mathcal {R}}{\mathbf {x}}_i = (\xi _i,\eta _i,\zeta _i)\in {\mathbb {R}}^3$, $1\le i\le k$, as data and seek a local reconstruction function of the form

$$\begin{aligned} \zeta = f(\xi ,\eta ). \end{aligned}$$

The function *f* can be any smooth function resulting from some interpolation or approximation/regression approach. We proceed to find *f* via a local least-squares polynomial reconstruction. For some $k>\text {dim}({\mathbb {P}}_n)$, where $\text {dim}({\mathbb {P}}_n)$ is the number of basis elements of the polynomial space ${\mathbb {P}}_p$ for the local surface reconstruction, the surface reconstruction function is obtained by solving the least-squares system

$$\begin{aligned} f:=\mathop {{{\,\mathrm{arg\,min}\,}}}\limits _{g\in {\mathbb {P}}_n}\sum _1^k\Vert {\zeta }_i - g(\xi _i,\eta _i)\Vert _2^2. \end{aligned}$$

Our *rotated* problem now is to identify the surface closest point of ${\mathcal {R}}{\mathbf {z}}$ to the surface given by $f(\xi ,\eta )$. We do so by solving a minimization problem on the reconstructed surface

11 $$\begin{aligned} (\xi _0,\eta _0) = \mathop {{{\,\mathrm{arg\,min}\,}}}\limits _{(\xi ,\eta )}\Vert {\mathcal {R}}{\mathbf {z}} - (\xi ,\eta ,f(\xi ,\eta ))\Vert _2^2, \end{aligned}$$

say, via some Newton-type iterative method with a good initial guess provided by the closest surface point ${\mathcal {R}}\mathbf {x_0}$ to the ${\mathcal {R}}{\mathbf {z}}$. The searching domain of (11) is specified by the convex hull formed by the first two coordinates in ${\mathcal {R}}X_k({\mathbf {z}})$.

**Note:** While Newton’s type methods for (11) with the good initial guess provided by ${\mathcal {R}}\mathbf {x_0}$ typically converge in 1 or 2 iterations, in case of non-convergence, either other techniques of mapping ${\mathcal {R}}{\mathbf {z}}$ to its closest point on the surface can be employed [13], or the contribution of the point ${\mathbf {z}}$ can be discarded as in [10].

The newly found closest point

$$\begin{aligned} cp_f({\mathcal {R}}{\mathbf {z}}) := f (\xi _0,\eta _0). \end{aligned}$$

should lie within the data points in ${\mathcal {R}}X_k({\mathbf {z}})$ used to define the local surface reconstruction. Finally, using the inverse rotation operator ${\mathcal {R}}^{-1}$, the new closest point is mapped back to the Cartesian coordinates to get the closest point on the surface $\varGamma$ via

$$\begin{aligned} cp_\varGamma ({\mathbf {z}}) = {\mathcal {R}}^{-1}cp_f({\mathcal {R}}{\mathbf {z}}). \end{aligned}$$

Note that the above is only one of the many possible approaches for approximating $cp_\varGamma ({\mathbf {z}})$; see [13] for an alternative surface reconstruction and minimizer identification. These local surface approximation approaches also allow one to identify other quantities such as the curvature, the unit normal or unit tangent vectors, as might be necessary.

### Surface Interpolations

An interpolation step is required in order to identify the solution value at some surface points after the use of the cp-projection in Sect. 3.2. There are numerous techniques that can be used, including meshfree or gridded interpolants. For non-smooth solutions, shock-capture interpolants can be used such as ENO or WENO schemes.

In the current work, we consider the interpolation technique defined in [12], that uses locally RBF-FD and an underlying grid. This interpolant allows for high order approximation of smooth solutions on smooth surfaces, and it can also be applied on non-uniform point distributions on the surface, thus exploring the aspects discussed in Sect. 3.2.

### Algorithmic Complexity

A breakdown of the floating point operations required for each of the algorithmic steps of Alg.1 at every time step using the scheme (8) is given in Table 1. Two different cases are considered for the analysis of the complexity of the algorithm: the case where the closest point function $cp_\varGamma$ is given analytically and the case where the framework defined in Sect. 3.2 is used for the identification of the closest points on point clouds. Given $cp_\varGamma$, the overall complexity of the algorithm is ${\mathcal {O}}(N)$, and the dominant cost lies at the choice of the interpolant (of stencil size *m*) in Alg.1 (b).

**Table 1:** The breakdown of the algorithmic complexity of each step of Alg.1 for the case of an analytic $cp_\varGamma$ and an approximation of the closest points using the framework in Sect.  3.2

Alg. Step	Alg.1 (a)	Alg.1 (b)	Alg.1 (c)
Analytic $cp_\varGamma$	${\mathcal {O}}(sN)$	${\mathcal {O}}(mN)$	${\mathcal {O}}(1)$
Computed $cp_\varGamma$	${\mathcal {O}}(sN\log (N))$	${\mathcal {O}}(mN)$	${\mathcal {O}}(1)$

For a computed $cp_\varGamma$, some additional computational work is required for Alg.1 (a). Using the framework in Sect. 3.2 and assuming a kd-tree structure for the storage of the points on the surface, a reduced QR solver for the local least squares fit and a Newton’s solver for the minimization problem, the construction of the kd-tree requires ${\mathcal {O}}(N\log (N))$ operations. This is a one-time cost that can be computed as a pre-processing step. The dominant cost is the search within the tree for each point and for each of the *s*-stages of the RK scheme, which requires ${\mathcal {O}}(sN\log (N))$ operations. The local surface reconstruction cost using direct solvers requires ${\mathcal {O}}(k\,dim({\mathbb {P}}_n)N)$ operations, where *k* is the number of points for local surface reconstruction and $dim({\mathbb {P}}_n)$ is the number of basis elements of the polynomial space ${\mathbb {P}}_p$ for the local surface reconstruction. Finally, the Newton type solver for the minimization process requires a total of ${\mathcal {O}}(N)$ operations for all points on the surface.

#### Remark 1

The framework in Sect. 3.2 is parallelizable, since the calculation of the closest point for each point is independent of the others.

#### Remark 2

The presented computational cost does not include the costs of the RBF-FD stencils from [12], since the algorithm is rather general and independent of the interpolant chosen for Alg.1 (b). The use of the RBF-FD stencils in [12] requires the use of another kd-tree requiring ${\mathcal {O}}(N\log (N))$ operations and can be performed as a pre-processing step. The solution of the local systems to estimate the RBF-FD stencil weights requires ${\mathcal {O}}((m+dim({\mathbb {P}}_p))^3N)$ operations using direct solvers. This calculation is parallelizable.

## Numerical Results

In the following numerical results, uniform Cartesian grids are considered with a spatial step-size $\varDelta x$. Unless stated otherwise, the minimum distance of the selected stencil $\delta _{\min }$ and the angle for consistent Lagrangian information $\theta _{\max }$ are $\sqrt{d}\varDelta x/5$ and $\pi /2$, respectively, where *d* is the dimension of the embedding space. The local surface fit uses quadratic polynomials with $k={\lceil }{2.5P}{\rceil }$ closest surface points (see Sect. 3.2), where *P* is the number of polynomial terms. The Newton method is employed to solve the minimization problem (11) for the identification of the new closest point on the surface with a relative error tolerance of $10^{-8}$.

The Polyharmonic Spline (PHS) RBF-FD interpolant is considered augmented with monomials that form a basis of the space ${\mathbb {P}}_p$ of order *p*, as defined in [12]. The stencil size is taken as $m={\lceil }{2.5q}{\rceil }$, where $q=\text {dim}({\mathbb {P}}_p)$ is the number of monomials that form the basis of ${\mathbb {P}}_p$ in ${\mathbb {R}}^d$.

### Unit Circle

To begin, we consider a simple example of a constant velocity ${\mathbf {v}} = {\mathbf {T}}$ on the unit circle, where ${\mathbf {T}}$ is the unit tangent vector. For this velocity, the solution of (5) for a smooth initial solution $u(0,s) = \sin (s)$, where *s* is the arclength of the circle, is given by

$$\begin{aligned} u(s,t) = \sin (s-t). \end{aligned}$$

**Fig. 2:**
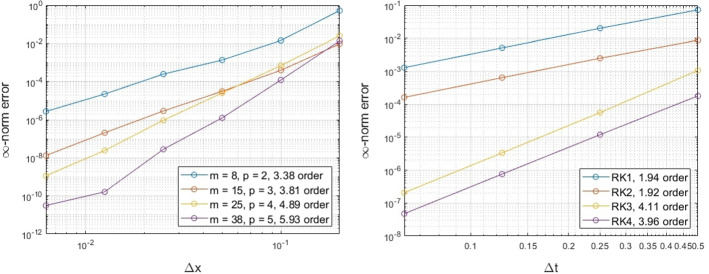
Exmp. 4.1: The spatial (left) and temporal (right) convergence of the proposed method on the unit circle (Color figure online)

We perform numerical simulations to explore both the spatial and the temporal convergence of the proposed method using different degrees *p* of the polynomial space ${\mathbb {P}}_p$ for the RBF-FD PHS interpolant, and different RK schemes, the forward Euler method (RK1), the midpoint method (RK2), Kutta’s third order method (RK3) and the classical fourth order method (RK4). For the temporal convergence, we use RK4 with a temporal step-size of $\varDelta t=0.01$, while for the spatial convergence we consider augmented polynomials that span ${\mathbb {P}}_4$ with a spatial step-size of $\varDelta x=0.01$. The $\infty$-norm relative error is considered to compare the numerical solution against the exact one. The results appear in Fig. 2.

The convergence rates appear to be consistent with the rates of the corresponding RBF-FD in the spatial convergence figure. Better than anticipated temporal convergence appears for the case of RK1 and RK3. This latter result arises due to the constant velocity chosen for this example, since some cancellation errors in these temporal discretization schemes lead to a higher order of convergence (see Appendix A.1.).

### Ellipse

Next, we consider the surface advection equation on an ellipse with minor axis $a=0.75$ on the x-axis and major axis $b=1.25$. Using a constant velocity ${\mathbf {v}}={\mathbf {T}}$ in the tangent direction, the exact solution at all times *t* is given by

$$\begin{aligned} u(s,t) = \sin (2\pi (s-t)/L), \end{aligned}$$

for the initial solution $u(s,0) = \sin (2\pi s/L)$, where *s* is the arclength and *L* is the circumference of the ellipse. Using a similar setup as the example on the unit circle, we explore the spatial and temporal convergence of our proposed method.

**Fig. 3:**
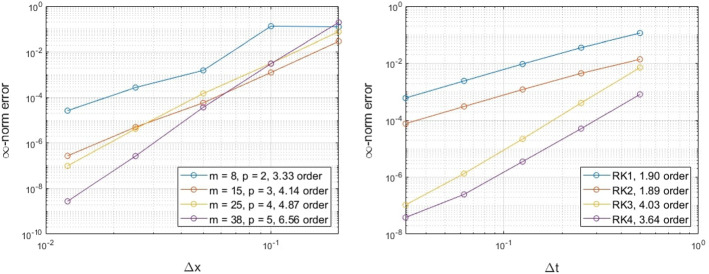
Exmp. 4.2: The spatial (left) and temporal (right) convergence of the proposed method on an ellipse (Color figure online)

The results in Fig. 3 appear to follow the expected convergence that is directed from the augmented polynomials of the RBF-FD scheme. A similar behavior as in the case of the unit circle appears for the temporal convergence, while a faster convergence appears for the cases of RK1 and RK3 (see Appendix A.1.).

### Unit Sphere

**Fig. 4:**
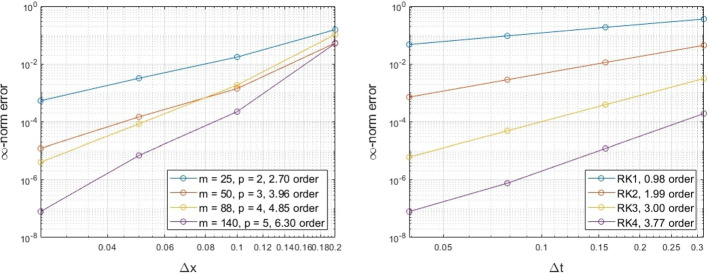
Exmp. 4.3: The spatial (left) and temporal (right) convergence of the proposed method on the unit sphere (Color figure online)

For our three dimensional examples, we consider an initial smooth solution

$$\begin{aligned} u(\theta ,\phi ,0) = \cos (\theta )\cos (\phi ), \end{aligned}$$

revolving around the unit sphere according to the velocity

$$\begin{aligned} {\mathbf {v}}(\theta ,\phi ) = \sin \theta \sin \phi {\mathbf {T}}_\theta + \cos \theta {\mathbf {T}}_\phi , \end{aligned}$$

where ${\mathbf {T}}_\theta$ and ${\mathbf {T}}_\phi$ are the unit tangent vectors that can be derived by differentiating the parametrized surface in the $\theta$ and $\phi$ parameters, respectively. The solution performs a full revolution around the sphere at time $2\pi$. We explore the spatial and temporal convergence of our method by comparing the numerical solution at the final time against the initial solution, using a fixed $\varDelta t = \pi /80$ and RK4 in the first case, and using the RBF-FD augmented with polynomials that span ${\mathbb {P}}_5$ with $\varDelta x = 0.025$ in the second case. The results appear in Fig. 4 and show the expected convergence, as dictated by the numerical discretizations in both space and time.

Another example on the unit sphere appears in [18], where a time dependent deformational flow ${\mathbf {v}}(\theta ,\phi ,t) = v_1{\mathbf {T}}_\theta + v_2{\mathbf {T}}_\phi$ is considered, where

$$\begin{aligned} v_1= & {} \frac{10}{T}\cos \left( \frac{\pi t}{T}\right) \sin ^2\left( \theta - \frac{2\pi t}{T}\right) \sin (2\phi ) +\frac{2\pi }{T}\cos \phi ,\\ v_2= & {} \frac{10}{T}\cos \left( \frac{2\pi t}{T}\right) \sin \left( 2\left( \theta - \frac{2\pi t}{T}\right) \right) \cos \phi . \end{aligned}$$

**Fig. 5:**
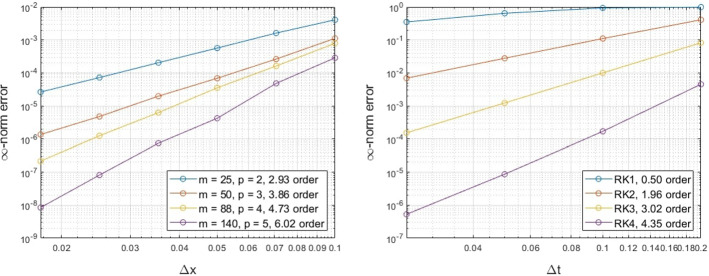
Exmp. 4.3: The spatial (left) and temporal (right) convergence of the proposed method on the unit sphere for a time dependent velocity (Color figure online)

The solution returns to its initial state at $T=5$. Given a smooth initial solution

$$\begin{aligned} u({\mathbf {x}},0) = 0.95\left( \exp ({-5\Vert {\mathbf {x}} - {\mathbf {q}}_1\Vert _2^2}) + \exp ({-5\Vert {\mathbf {x}} - {\mathbf {q}}_2\Vert _2^2})\right) \end{aligned}$$

the spatial and temporal convergence of the numerical method appears in Fig. 5. For the temporal convergence, we consider a fixed spatial step-size $\varDelta x = 0.025$ and the PHS RBF-FD with augmented polynomials that span ${\mathbb {P}}_5$. In addition, a small, fixed time step-size of $\varDelta t = 0.00625$ is considered with the RK4 scheme in order to observe only the spatial errors.

The results show that we get the expected convergence for the methods considered in both the spatial and temporal convergence tests.

### Torus

In this example, we consider a torus with outer radius $R=1$ and inner radius $r = 1/3$ parametrized as

$$\begin{aligned} {\mathbf {x}}(\theta ,\phi ) = ((R+r\cos \phi )\cos \theta ,(R+r\cos \phi )\sin \theta ,r\sin \phi ). \end{aligned}$$

**Fig. 6:**
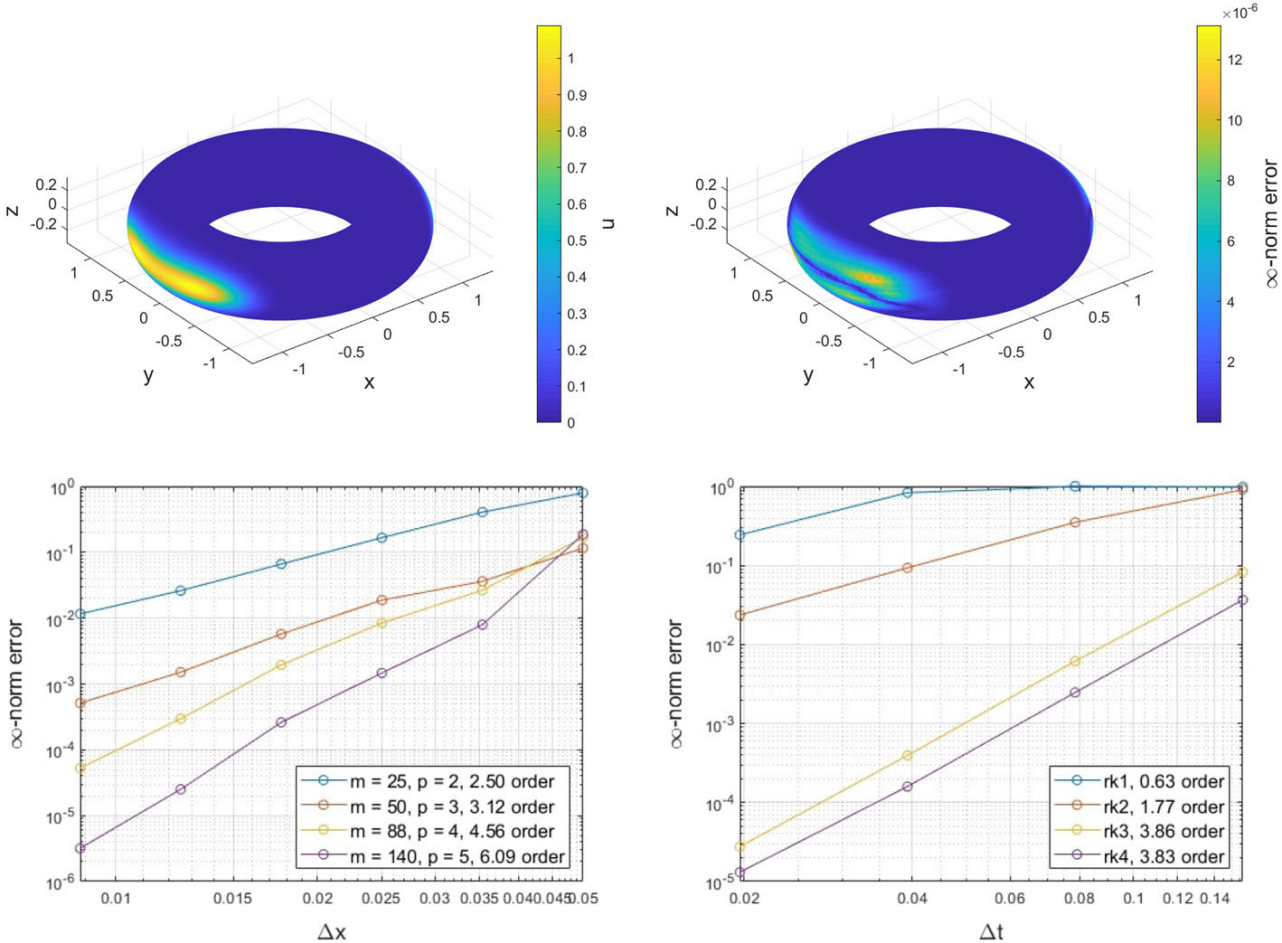
Exmp. 4.4: The initial solution on the torus (top left) and the $\infty$-norm relative error against the initial solution (top right) at the final time. The spatial (bottom left) and temporal (bottom right) convergence of the proposed method on a torus for the (3,2) torus knot velocity (Color figure online)

Following [19], a torus knot (3,2) is considered for the velocity, defined as

$$\begin{aligned} v_x= & {} \rho _2\cos (3\varTheta ) - 3\rho _1\sin (3\varTheta ), \\ v_y= & {} \rho _2\sin (3\varTheta ) + 3\rho _1\cos (3\varTheta ), \\ v_z= & {} 2r\cos (2\varPhi ), \end{aligned}$$

where

$$\begin{aligned} \rho = \sqrt{x^2+y^2},\qquad \varTheta = \frac{1}{3}\tan ^{-1}\left( \frac{y}{x}\right) ,\qquad \varPhi = \frac{1}{2}\tan ^{-1}\left( \frac{z}{\rho -R}\right) , \end{aligned}$$

and $\rho _1 = R+r\cos (2\varPhi )$, $\rho _2 = -2r\sin (2\varPhi )$. In the notation above, ${\mathbf {x}} = (x,y,z)$ and ${\mathbf {v}} = (v_x,v_y,v_z)$. The solution returns to the initial position after a time $T=2\pi$. For an initial solution

$$\begin{aligned} u({\mathbf {x}},0) = \exp ({-a(x-q_1)^2+y^2-1.5az^2})+\exp ({-a(x-q_2)^2+y^2-1.5az^2}), \end{aligned}$$

where $a=20$, $q_1=1+1/3$ and $q_2 = -q_1$, the results for the spatial and temporal convergence appear in Fig. 6.

We compare the numerical results at a full revolution against the initial solution using a time step-size of $\varDelta t=\pi /160$ and the RK4 scheme for the spatial convergence, and a $\varDelta x=0.0125$ and augmented polynomials in the RBF-FD schemes that span ${\mathbb {P}}_5$ for the temporal one. As expected, the spatial convergence rates agree with the order of the corresponding augmented polynomial terms of the RBF-FD chosen. A faster convergence is obtained for RK1 and RK3, possibly due to a cancellation error in the temporal discretization scheme (see Appendix A). The relative error distribution on the torus at final time for the RK4 case with $\varDelta t=\pi /160$, $\varDelta x=0.0125$ and augmented polynomials in the RBF-FD schemes that span ${\mathbb {P}}_5$ appears in Fig. 6.

## Extension to General PDEs

The algorithm for more general PDEs considers two main classes of problems: *weakly coupled systems*, where the velocity ${\mathbf {v}}$ is independent of the solution *u*, and *strongly coupled systems*, where the velocity ${\mathbf {v}}$ depends on the solution *u*. For a point cloud $Y = \{{\mathbf {y}}_j\}_j\subset \varGamma$, the algorithmic steps for weakly coupled systems follow in Alg.2:

- **Identify the backtraced points:** Use step Alg.1(a) of the algorithm presented in Sect. 3.1 to approximate the backtraced points $\tilde{{\mathbf {z}}}(t_n)$ and store $cp_\varGamma (\tilde{{\mathbf {z}}}(t_{n}))=:{\mathbf {x}}_j$.
- **Interpolate the solution on the backtraced points:** Use step Alg.1(b) to obtain ${\tilde{u}}_\varOmega ({\mathbf {x}}_j)$.
- **Solve the PDE:** Using a standard numerical discretization method over the time interval $[t_n,t_{n+1}]$, solve the PDE on $\{{\mathbf {x}}_j\}_j$ to obtain the updated $\{{\tilde{u}}_\varOmega ({\mathbf {x}}_j)\}_j$.
- **Update the nodal value:** Use step Alg.1(c) to obtain $u_\varOmega ({\mathbf {y}}_j,t_{n+1})$.

**Note:** Alg.1 describes a framework for the solution of PDE systems of the form

$$\begin{aligned} \frac{Du}{Dt} = 0, \end{aligned}$$

where $D(\cdot )/Dt$ is the material derivative, while Alg.2 is an extension to more general PDEs of the form

$$\begin{aligned} \frac{Du}{Dt} = F(u({\mathbf {x}},t),{\mathbf {v}}({\mathbf {x}},t),{\mathbf {x}},t), \end{aligned}$$

where *F* denotes some general right-hand side function.

Unlike the previous two algorithms, in the strongly coupled system case the backtracing of the surface nodes requires rigorous techniques such as [4, 16] that are out of the scope of this paper due to the nature of the problem, where the velocity depends on the solution of the PDE. Thus, a forward tracing is carried out, as described in Alg.3.

- **Solve the PDE:** Using a standard numerical discretization method over the time interval $[t_n,t_{n+1}]$, solve the PDE on $\{{\mathbf {y}}_j\}_j$ to obtain $\{{\tilde{u}}_\varOmega ({\mathbf {y}}_j)\}_j$.
- **Identify the forward traced points:** For each surface point ${\mathbf {y}}_j\in Y$ solve the ODE
  12 $$\begin{aligned} \begin{aligned}&\frac{d}{dt}cp_\varGamma ({\mathbf {z}}(t)) = {\mathbf {v}}(cp_\varGamma ({\mathbf {z}}(t)),t), \\&{\mathbf {z}}(t_{n}) = {\mathbf {y}}_j, \end{aligned} \end{aligned}$$
  over the time interval $[t_n,t_{n+1}]$ to identify the forward traced points $\tilde{{\mathbf {z}}}(t_{n+1})$ and store $cp_\varGamma (\tilde{{\mathbf {z}}}(t_{n+1}))=:{\mathbf {x}}_j$. The solution is carried to the forward traced nodes, i.e., ${\tilde{u}}({\mathbf {x}}_j)={\tilde{u}}({\mathbf {y}}_j)$.
- **Interpolate the solution on the initial point cloud:** Use existing nodal solution values $\{({\mathbf {x}}_j,{\tilde{u}}_\varOmega ({\mathbf {x}}_j))\}$; as data to interpolate the solution ${\tilde{u}}_\varOmega$ to the initial point cloud $\{{\mathbf {y}}_j\}_j$, obtaining the nodal solution $u_\varOmega ({\mathbf {y}}_j,t_{n+1})$.

Note that even for strongly coupled systems, Alg.3(c) guarantees that the solution is mapped back to the initial point cloud *Y*.

### Non-Solenoidal Velocity

In this section, we focus on Eq. (1) with some non-solenoidal velocity fields either on the unit circle or the unit sphere, namely

13 $$\begin{aligned} \left\{ \begin{array}{r@{~=~}l} \partial _t u + {\mathbf {v}}\varvec{\cdot }\nabla _\varGamma u &{} -u\nabla _\varGamma \varvec{\cdot }{\mathbf {v}}, \\ u(\cdot , 0) &{} u_0, \end{array} \right. \quad \text{ on } \varGamma . \end{aligned}$$

Alg. 2 is used to numerically approximate this weakly coupled system. Unless stated otherwise, the same RK scheme is considered for both the solution of the ODE and the solution of the PDE in Alg.2.

#### Unit Circle

We consider an example of a surface advection PDE with a non-solenoidal spatial dependent velocity

$$\begin{aligned} {\mathbf {v}} = (1+\cos (s)){\mathbf {T}}. \end{aligned}$$

Using the same initial solution as in Sect. 4.1, $u(0,s) = \sin (s)$, the solution is

$$\begin{aligned} u(s,t) = \sin (\xi (s,t))\frac{(c(s,t)+t)^2+1}{c^2(s,t)+1}, \end{aligned}$$

where

$$\begin{aligned} \xi (s,t) = -2\tan ^{-1}(t - \tan (s/2))\qquad \text {and}\qquad c(s,t) = \tan (\xi (s,t)/2). \end{aligned}$$

**Fig. 7:**
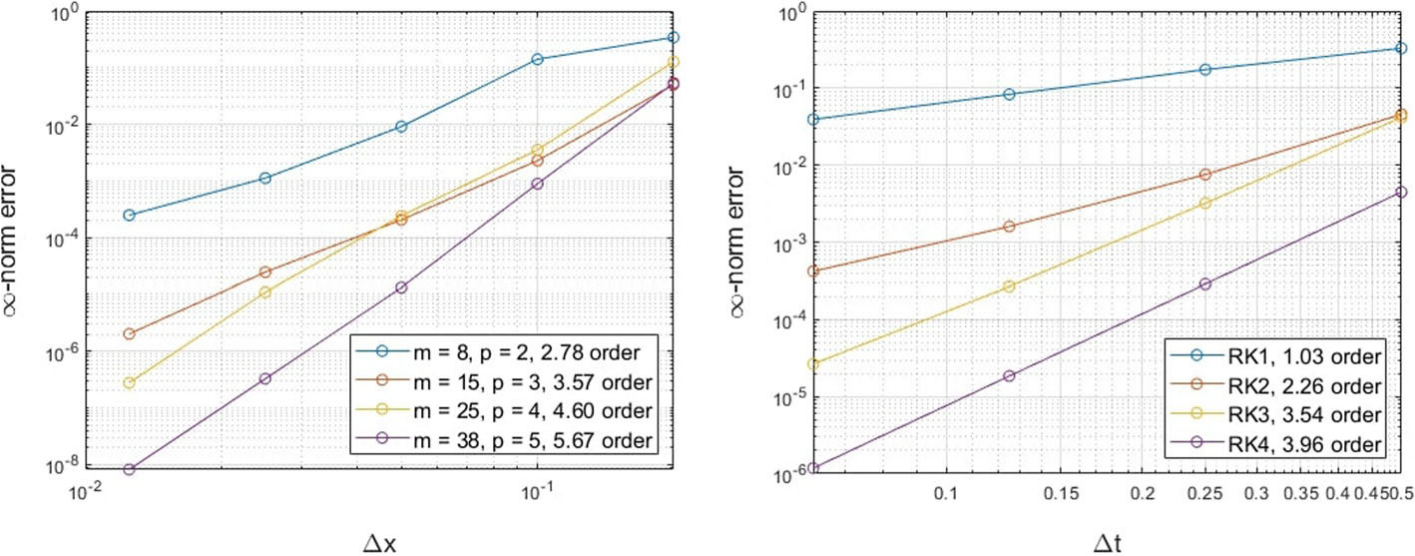
Exmp. 5.1.1: The spatial (left) and temporal (right) convergence of the proposed method on the unit circle for a spatial dependent velocity (Color figure online)

The results appear in Fig. 7. The temporal convergence appears to follow the corresponding theoretical rates of the RK methods and the observed spatial convergence agrees with the expected rates.

In order to obtain the expected convergence rates shown for the cases of RK3 and RK4, the use of an approximation for the midpoint ${\mathbf {x}}_j^{n+1/2}$ that is at most one order of accuracy less than the approximation of ${\mathbf {x}}_j^n$ is required in Alg.2(a) of Sect. 3.1. In particular, in the case of RK3 for the approximation of the backtraced points of ${\mathbf {x}}_j^n$, we use RK2 for the approximation of the backtraced midpoint ${\mathbf {x}}_j^{n+1/2}$, and in the case of RK4 we use RK3 for the midpoint. The use of lower order methods to approximate the midpoints was found to reduce the convergence order of Alg.2 for both RK3 and RK4.

#### Unit Sphere

**Fig. 8:**
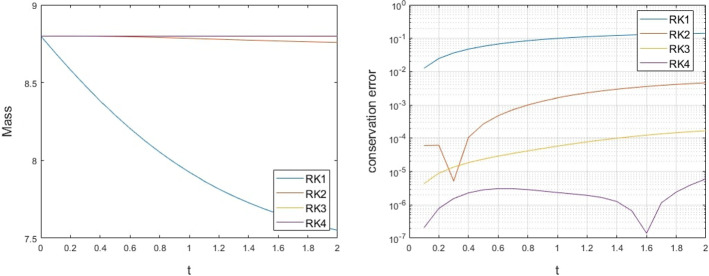
Exmp. 5.1.2: The mass conservation of the solution (left) and the error of the mass conservation (right) on the unit sphere along time (Color figure online)

In our next example, consider the Eq. (2) on the unit sphere, with the non-solenoidal velocity

$$\begin{aligned} {\mathbf {v}}(\theta ,\phi ) = \sin \theta \sin \phi {\mathbf {T}}_\theta + (\cos \theta +\cos \phi ){\mathbf {T}}_\phi , \end{aligned}$$

for which clearly $\nabla _\varGamma \cdot {\mathbf {v}}\ne 0$. Using the initial solution

$$\begin{aligned} u(\theta ,\phi ,0) = \cos (\theta )\cos (\phi ), \end{aligned}$$

the evolution of the solution is numerically approximated with the proposed framework. The solution is approximated until the final time $t = 2$ with a time step-size $\varDelta t=0.1$, and the conservation of the solution is estimated by the second-order integration method presented in [9]. Using a spatial discretization of $\varDelta x=0.025$ and RBF-FD augmented with polynomials that span ${\mathbb {P}}_4$, the conservation of the solution over time and the error in the conserved initial mass appear in Fig. 8.

The conservation of the solution improves as the order of the RK method increases, with RK4 providing the best conservation results in time. Similar to the non-solenoidal example on the unit circle, we use RK2 for the approximation of the midpoint in the RK3 case and RK3 in the RK4 case.

### Inviscid Burgers’ Equation

The next example employs the surface inviscid Burgers’ equation, given as

$$\begin{aligned} \partial _t u + {\mathbf {v}}\varvec{\cdot }\nabla _\varGamma u = 0, \end{aligned}$$

with ${\mathbf {v}} = u{\mathbf {T}}$, where ${\mathbf {T}}$ is the unit normal vector. In this example, we consider that $\varGamma$ is the unit circle. Using the method of characteristics, the solution *u* is constant along the characteristics, which are given as

$$\begin{aligned} \theta (t) = u_0(\xi )t+\xi , \end{aligned}$$

where $-\pi \le \theta \le \pi$ is the arclength of the circle, $\xi =\theta (0)$, $u_0(\xi )$ is the initial solution and *t* is the time. The exact solution is given implicitly as

$$\begin{aligned} u(\theta ,t) = u_0(u_0(\xi )t + \xi ). \end{aligned}$$

Since this is a strongly coupled system, we use the algorithm Alg.3 in Sect. 5. The interpolation step is applied as indicated in the algorithm, however we apply the technique presented in [5] using the PHS RBF-FD augmented with polynomials that span ${\mathbb {P}}_p$. Any other meshfree interpolant could be used for this example, since the forward evolution in the algorithm does not allow the use of an interpolant that requires a grid.

Given an initial solution

$$\begin{aligned} u_0(\theta ) = \exp ({-\theta ^2}), \end{aligned}$$

**Fig. 9:**
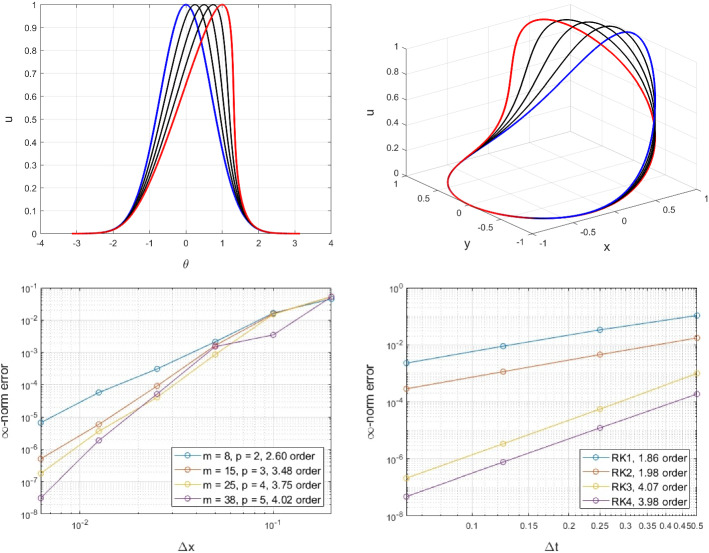
Exmp. 5.2: The solution at the initial time (in blue) and the transition (in black) to the final time (in red) in 2D (top left) and 3D (top right). The spatial (bottom left) and temporal (bottom right) convergence of the proposed method on the unit circle for the inviscid Burger’s equation (Color figure online)

Fig. 9 shows the spatial and temporal convergence of the method at the final time of $t=1$. This corresponds to an evolution before the first shock occurrence at $T_c = \sqrt{e/2}$. When a shock discontinuity occurs, the inviscid Burgers’ equation does not have a unique solution and an entropy condition is required for the identification of the correct weak solution. Typical numerical schemes fail to accurately capture the speed of the shock, and thus cannot be convergent [20]. The exploration of numerical methods for the accurate capture of the shock solution is beyond the scope of this paper.

We obtain a faster than expected convergence for the RK1 and RK3 schemes, since a cancellation error leads to higher order of convergence (see Appendix A.2.). The spatial convergence follows the expected rates dictated from the augmented polynomials in the RBF-FD stencils.

### Reaction-Advection-Diffusion Equation

Our final example considers the reaction-diffusion Gray-Scott system of equations with an advection term, given as

14 $$\begin{aligned} \begin{aligned} u_t + \nabla _\varGamma \varvec{\cdot }({\mathbf {v}} u)&= f(u,w) + \nu _u\varDelta _\varGamma u,\\ w_t + \nabla _\varGamma \varvec{\cdot }({\mathbf {v}} w)&= g(u,w) + \nu _w\varDelta _\varGamma w, \end{aligned} \end{aligned}$$

where ${\mathbf {v}}$ is the velocity field, *u* and *w* are the concentrations of the chemicals, *f* and *g* are the mixing functions and $\nu _u$ and $\nu _w$ are the diffusion rates. The mixing functions are given as

$$\begin{aligned} f(u,w)= & {} -uw^2 + F(1-u), \\ g(u,w)= & {} uw^2 - (F+k)w, \end{aligned}$$

where *F* and *k* are constants. The PDE is considered on four different surfaces: the unit sphere $(\varGamma _1)$, the torus from the example in Sect. 4.4$(\varGamma _2)$, the Dziuk surface $(\varGamma _3)$ given as an implicit surface

$$\begin{aligned} (x-z^2)^2+y^2+z^2=1, \end{aligned}$$

and the torus link 1 surface $(\varGamma _4)$ given as a parametrized surface

$$\begin{aligned} {\mathbf {x}}(\theta ,\phi ) = ((R+r\rho (\theta ,\phi ))\cos (2\theta ),(R+r\rho (\theta ,\phi ))\sin (2\theta ), r(r_z\sin (2\phi )+\sin \theta )), \end{aligned}$$

where

$$\begin{aligned} \rho (\theta ,\phi ) = r_z\cos (2\phi ) + \cos \theta , \end{aligned}$$

and $$-\pi \le \theta ,\phi \le \pi $$, $$R=1$$, $$r=0.25$$ and $$r_z=0.7$$.

The velocities considered are given as

$$\begin{aligned}&{\mathbf {v}}_{\varGamma _1}(\theta ,\phi ) = 0.2(\sin \theta \sin \phi {\mathbf {T}}_\theta + \cos \theta {\mathbf {T}}_\phi ),\\&{\mathbf {v}}_{\varGamma _2}(\varTheta ,\varPhi ) = 0.1(\rho _2\cos (3\varTheta ) - 3\rho _1\sin (3\varTheta ), \rho _2\sin (3\varTheta ) + 3\rho _1\cos (3\varTheta ), 2r\cos (2\varPhi )),\\&{\mathbf {v}}_{\varGamma _3} = 0.1({\mathbf {v}}_1-({\mathbf {v}}_1\varvec{\cdot }{\mathbf {n}}){\mathbf {n}}),\\&{\mathbf {v}}_{\varGamma _4} = 0.1{\mathbf {n}}\times (-y,x,0),\\ \end{aligned}$$

for each surface, where ${\mathbf {v}}_1$ is the spatially dependent velocity in the example in Sect. 4.3 scaled by 0.2 and ${\mathbf {v}}_2$ is the velocity in 4.4 scaled by 0.1. In all cases the velocity is solenoidal, i.e., $\nabla _\varGamma \varvec{\cdot }{\mathbf {v}}_{\varGamma _i}=0$, for $i = 1,\ldots ,4$.

**Fig. 10:**
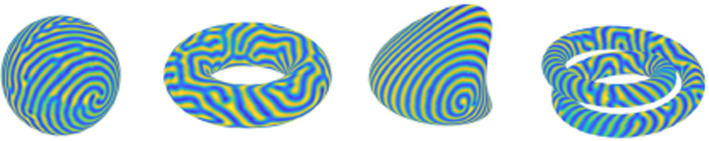
Exmp. 5.3: The stripe patterns of the solution of the reaction-advection-diffusion system on the unit sphere, the torus, the Dziuk surface and the torus link 1 surface

Using the parameter set $F=0.054$, $k=0.063$, $\nu _u=(\varDelta x/3)^2$ and $\nu _v = \nu _u/3$, the resulting stripe patterns appear in Fig. 10. For the examples, the RK2 scheme for the temporal discretization of the method of characteristics and the Forward Euler scheme for the solution of the ODE system for *u* and *w* are considered. The PHS RBF-FD augmented with polynomials that span ${\mathbb {P}}_4$ are employed, as presented in [12]. We select the time step-size as $\varDelta t = 0.1 \varDelta x^2/\nu _u$, with the spatial step-size $\varDelta x = 0.025$. The Dziuk surface has high curvature areas and the torus link 1 has segments that are close to each other, thus we use a consistency condition on the normal vectors when selecting the local RBF-FD stencils, similar to the one presented in Sect. 3.2. Note that in all cases the stripes patterns align with the corresponding velocity field in Fig. 10.

## Conclusion

In this paper, a semi-Lagrangian numerical framework for solving advection conservation laws and advection dominated PDEs is presented. The framework uses the projection method of Sect. 3 in combination with the closest point mapping and interpolation to track the particle motion and advance the solution. The method is mesh independent and can be used as part of general frameworks for point clouds. For the case where the closest point mapping is not given, a point cloud based framework is presented, where local surface reconstruction allows for the identification of the surface closest points. The method is proved to be consistent with the PDE defined in the embedded space of the surface. Numerical results show the convergence of the method for advection conservation laws with solenoidal velocity fields, in which the expected convergence rates are obtained.

An extension of the framework to general PDEs is also presented, where the semi-Lagrangian method is considered as part of the solution of rather general PDEs. Numerical examples include advection conservation laws with non-solenoidal velocities, the inviscid Burger’s equation and reaction-advection-diffusion systems on rather general surfaces, including surfaces with high curvature areas and with different surface segments being close to one another.

The numerical framework is tested on smooth surfaces using smooth solutions. Further work is required for the solution of advection conservation laws with non-smooth solutions, where numerical schemes such as ENO/WENO should be employed. Another interesting aspect is the generalization of this framework to moving surfaces. A similar approach has been used in [2] for moving triangulated surfaces, however no work has been presented for moving point clouds. These future directions are part of our ongoing research.

## Data Availability

Not applicable.

## Appendix A. Error estimation using RK1

We are interested in the calculation of the error of a function *u* carried along the trajectories ${\mathbf {x}}(t)$. Assume a PDE of the form

$$\begin{aligned} \frac{Du}{Dt} = u_t + {\mathbf {v}}\cdot \nabla _\varGamma u = 0,\qquad {\mathbf {v}} = {\mathbf {v}}({\mathbf {x}},t),\qquad {\mathbf {x}}\in \varGamma \end{aligned}$$

on a surface $\varGamma$. Using a semi-Lagrangian approach, the trajectories ${\mathbf {x}}(t)$ on which the solution *u* should be constant follow the ODE

$$\begin{aligned} \frac{d{\mathbf {x}}}{dt} = {\mathbf {v}}. \end{aligned}$$

Let us assume that we are interested in the calculation of the error at time $t_{n+1}=t_n+\varDelta t$ using the RK1 scheme. Then, for a fixed ${\mathbf {x}}(0)$ at $t_0=0$, we evaluate the local time stepping error by introducing the RK1 scheme for the estimation of ${\mathbf {x}}(t_{n+1})$

$$\begin{aligned} {\mathbf {x}}(t_{n+1}) \approx {\mathbf {x}}(t_n) + \varDelta t{\mathbf {v}}(t_n,{\mathbf {x}}(t_n)). \end{aligned}$$

into the following term which evaluates to zero analytically

$$\begin{aligned} \Vert u(t_{n+1},{\mathbf {x}}(t_{n+1}))-u(0,{\mathbf {x}}(0))\Vert . \end{aligned}$$

We assume infinitely smooth functions *u* and ${\mathbf {v}}$ and apply a Taylor series expansion around $t_n$ and ${\mathbf {x}}(t_n)$ to get

$$\begin{aligned} u(t_{n+1},{\mathbf {x}}(t_n) + \varDelta t{\mathbf {v}}(t_n,{\mathbf {x}}(t_n))) = u(t_n,{\mathbf {x}}(t_n)) + F_1\varDelta t + F_2\frac{\varDelta t^2}{2} + {\mathcal {O}}(\varDelta t^3). \end{aligned}$$

Let us calculate the terms of the series:

$$\begin{aligned} F_1= & {} u_t + {\mathbf {v}}\cdot \nabla _\varGamma u = 0 \text { (from PDE)} \\ F_2= & {} u_{tt} + 2{\mathbf {v}}\cdot \nabla _\varGamma u_t + {\mathbf {v}}^T(\nabla _\varGamma (\nabla _\varGamma u)){\mathbf {v}}\text { (diff. PDE for }t\text { and sub.)} \\= & {} -\frac{\partial {\mathbf {v}}}{\partial t}\cdot \nabla _\varGamma u - {\mathbf {v}}\cdot \nabla _\varGamma ({\mathbf {v}}\cdot \nabla _\varGamma u) + {\mathbf {v}}^T(\nabla _\varGamma (\nabla _\varGamma u)){\mathbf {v}} \text { (sub. } u_t\text { from PDE)} \end{aligned}$$

Thus, for a velocity that satisfies the condition

$$\begin{aligned} -\frac{\partial {\mathbf {v}}}{\partial t}\cdot \nabla _\varGamma u - {\mathbf {v}}\cdot \nabla _\varGamma ({\mathbf {v}}\cdot \nabla _\varGamma u) + {\mathbf {v}}^T(\nabla _\varGamma (\nabla _\varGamma u)){\mathbf {v}}=0 \end{aligned}$$

our approximation procedure leads to an ${\mathcal {O}}(\varDelta t^2)$ error:

$$\begin{aligned} \Vert u(t_{n+1},{\mathbf {x}}(t_{n+1})) - u(0,{\mathbf {x}}(0))\Vert\approx & {} \Vert u(t_{n+1},{\mathbf {x}}(t_n) + \varDelta t{\mathbf {v}}(t_n,{\mathbf {x}}(t_n)))-u(0,{\mathbf {x}}(0))\Vert \\\le & {} \Vert u(t_n,{\mathbf {x}}(t_n)) - u(0,{\mathbf {x}}(0))\Vert + {\mathcal {O}}(\varDelta t^3) \\\approx & {} \Vert u(t_n,{\mathbf {x}}(t_{n-1}) + \varDelta t{\mathbf {v}}(t_{n-1},{\mathbf {x}}(t_{n-1})))\\&-u(0,{\mathbf {x}}(0))\Vert + {\mathcal {O}}(\varDelta t^3) \\\le & {} \Vert u(t_{n-1},{\mathbf {x}}(t_{n-1})) - u(0,{\mathbf {x}}(0))\Vert + 2{\mathcal {O}}(\varDelta t^3) \\\le & {} \ldots \le \\\le & {} (n+1){\mathcal {O}}(\varDelta t^3) = {\mathcal {O}}(\varDelta t^2) \end{aligned}$$

### Appendix A.1. Constant speed along trajectory case

Let us consider the special case of the constant speed along a continuous and smooth unit tangent vector field. In the case of smooth curves in ${\mathbb {R}}^2$, an example of such velocity for a unit tangent vector ${\mathbf {T}}$ takes the form

$$\begin{aligned} {\mathbf {v}} = c{\mathbf {T}}, \end{aligned}$$

for some $c\in {\mathbb {R}}$. Assuming an arc-length *s* for the curve, we get

$$\begin{aligned} \frac{\partial {\mathbf {v}}}{\partial t} = 0\qquad \text {and}\qquad {\mathbf {v}}\cdot \nabla _\varGamma ({\mathbf {v}}\cdot \nabla _\varGamma u) = {\mathbf {v}}^T(\nabla _\varGamma (\nabla _\varGamma u)){\mathbf {v}}=c^2\frac{\partial ^2 u}{\partial s^2}. \end{aligned}$$

Thus, $F_2=0$ and so each step of size $\varDelta t$ using the RK1 scheme yields an error of ${\mathcal {O}}(\varDelta t^3)$ for the numerical approximation of the solution *u*.

On the other hand, for a smooth surface $\varGamma$ in ${\mathbb {R}}^3$, let ${\mathbf {T}}_1$ and ${\mathbf {T}}_2$ be its orthonormal tangential vectors, which are continuous along $\varGamma$. Then, for a velocity defined as

$$\begin{aligned} {\mathbf {v}} = c_1{\mathbf {T}}_1+c_2{\mathbf {T}}_2, \end{aligned}$$

where $c_1,c_2\in {\mathbb {R}}$ we get

$$\begin{aligned} F_2= & {} c^2_1\left( {\mathbf {T}}_1\cdot \left( \frac{\partial {\mathbf {T}}_1}{\partial {\mathbf {T}}_1} + \frac{\partial {\mathbf {T}}_2}{\partial {\mathbf {T}}_1}\right) \right) \frac{\partial u}{\partial {\mathbf {T}}_1} + c^2_2\left( {\mathbf {T}}_2\cdot \left( \frac{\partial {\mathbf {T}}_1}{\partial {\mathbf {T}}_2} + \frac{\partial {\mathbf {T}}_2}{\partial {\mathbf {T}}_2}\right) \right) \frac{\partial u}{\partial {\mathbf {T}}_2} \\&+\, c_1c_2\left[ ({\mathbf {T}}_1+{\mathbf {T}}_2)\cdot \frac{\partial {\mathbf {T}}_1}{\partial {\mathbf {T}}_1}\frac{\partial u}{\partial {\mathbf {T}}_1} + {\mathbf {T}}_1\cdot \frac{\partial {\mathbf {T}}_1}{\partial {\mathbf {T}}_2}\frac{\partial u}{\partial {\mathbf {T}}_1} + {\mathbf {T}}_2\cdot \frac{\partial {\mathbf {T}}_2}{\partial {\mathbf {T}}_1}\frac{\partial u}{\partial {\mathbf {T}}_2} \right. \\&\left. +\, ({\mathbf {T}}_1+{\mathbf {T}}_2)\cdot \frac{\partial {\mathbf {T}}_2}{\partial {\mathbf {T}}_2}\frac{\partial u}{\partial {\mathbf {T}}_2}\right] \ne 0. \end{aligned}$$

### Appendix A.2. Inviscid Burgers’ equation case

Consider the inviscid Burgers’ equation defined on a smooth curve $\varGamma \subset {\mathbb {R}}^2$

$$\begin{aligned} u_t + {\mathbf {v}}\cdot \nabla _\varGamma u = 0, \qquad {\mathbf {x}}\in \varGamma \end{aligned}$$

where ${\mathbf {v}} = u{\mathbf {T}}$. We can write the semi-Lagrangian system as

$$\begin{aligned} \frac{d{\mathbf {x}}}{dt} = u(t,{\mathbf {x}}(t)){\mathbf {T}}, \end{aligned}$$

where ${\mathbf {T}}$ is the continuous unit tangent vector and *u* is a function for which $u(t,{\mathbf {x}}(t)) = u(0,{\mathbf {x}}(0)) = u_0$. Thus, we can rewrite the ODE as

$$\begin{aligned} \frac{d{\mathbf {x}}}{dt} = u_0{\mathbf {T}}. \end{aligned}$$

Let us use a similar analysis to our previous one to estimate the Taylor expansion terms for the Burgers’ equation for a fixed ${\mathbf {x}}(0)$:

$$\begin{aligned} F_1= & {} u_t + {\mathbf {v}}\cdot \nabla _\varGamma u = 0 \text { (from PDE)} \\ F_2= & {} u_{tt} + 2{\mathbf {v}}\cdot \nabla _\varGamma u_t + {\mathbf {v}}^T(\nabla _\varGamma (\nabla _\varGamma u)){\mathbf {v}}\text { (diff. PDE for }t\text { and sub.)} \\= & {} u({\mathbf {T}}\cdot \nabla _\varGamma u)^2 - u{\mathbf {T}}\cdot \nabla _\varGamma (u{\mathbf {T}}\cdot \nabla _\varGamma u) + u^2{\mathbf {T}}^T(\nabla _\varGamma (\nabla _\varGamma u)){\mathbf {T}} \text { (sub. }u_t\text { from PDE)} \end{aligned}$$

Let *s* be the arc-length of the curve $\varGamma$. Then, we can write

$$\begin{aligned} F_2 = u\left( \frac{\partial u}{\partial s}\right) ^2 - u\frac{\partial }{\partial s}\left( u\frac{\partial u}{\partial s}\right) +u^2\frac{\partial ^2u}{\partial s^2}=0. \end{aligned}$$

Thus each step of size $\varDelta t$ using the RK1 scheme yields a local error of ${\mathcal {O}}(\varDelta t^3)$ for the numerical approximation of the solution *u*.

On the other hand, assuming a smooth surface $\varGamma$ with its orthonormal tangential vectors ${\mathbf {T}}_1$ and ${\mathbf {T}}_2$, that define a continuous vector field along the surface, the inviscid Burgers’ equation can be written as

$$\begin{aligned} u_t + {\mathbf {v}}\cdot \nabla _\varGamma u= & {} 0, \\ v_t + {\mathbf {v}}\cdot \nabla _\varGamma v= & {} 0, \end{aligned}$$

where the velocity is defined as

$$\begin{aligned} {\mathbf {v}} = u{\mathbf {T}}_1+v{\mathbf {T}}_2. \end{aligned}$$

Similar to the previous case, using the Taylor expansion for *u* we get

$$\begin{aligned} F_2= & {} u^2\left[ {\mathbf {T}}_1\cdot \left( \frac{\partial {\mathbf {T}}_1}{\partial {\mathbf {T}}_1} + \frac{\partial {\mathbf {T}}_2}{\partial {\mathbf {T}}_1}\right) \frac{\partial u}{\partial {\mathbf {T}}_1}\right] + v^2\left[ {\mathbf {T}}_2\cdot \left( \frac{\partial {\mathbf {T}}_1}{\partial {\mathbf {T}}_2} + \frac{\partial {\mathbf {T}}_2}{\partial {\mathbf {T}}_2}\right) \frac{\partial u}{\partial {\mathbf {T}}_2}\right] \\&+\, uv\left[ ({\mathbf {T}}_1+{\mathbf {T}}_2)\cdot \frac{\partial {\mathbf {T}}_1}{\partial {\mathbf {T}}_1}\frac{\partial u}{\partial {\mathbf {T}}_1} + {\mathbf {T}}_1\cdot \frac{\partial {\mathbf {T}}_1}{\partial {\mathbf {T}}_2}\frac{\partial u}{\partial {\mathbf {T}}_1} + {\mathbf {T}}_2\cdot \frac{\partial {\mathbf {T}}_2}{\partial {\mathbf {T}}_1}\frac{\partial u}{\partial {\mathbf {T}}_2} \right. \\&\left. +\, ({\mathbf {T}}_1+{\mathbf {T}}_2)\cdot \frac{\partial {\mathbf {T}}_2}{\partial {\mathbf {T}}_2}\frac{\partial u}{\partial {\mathbf {T}}_2}\right] \ne 0. \end{aligned}$$

An analogous result can be obtained for *v*, indicating that each step of size $\varDelta t$ using the RK1 scheme yields an error of ${\mathcal {O}}(\varDelta t^2)$ for the numerical approximation of the solutions *u* and *v*.

## Code Availability

The code is available upon request to the corresponding author.
